# Potential and Applications of Nanocarriers for Efficient Delivery of Biopharmaceuticals

**DOI:** 10.3390/pharmaceutics12121184

**Published:** 2020-12-06

**Authors:** Alam Zeb, Isra Rana, Ho-Ik Choi, Cheol-Ho Lee, Seong-Woong Baek, Chang-Wan Lim, Namrah Khan, Sadia Tabassam Arif, Najam us Sahar, Arooj Mohsin Alvi, Fawad Ali Shah, Fakhar ud Din, Ok-Nam Bae, Jeong-Sook Park, Jin-Ki Kim

**Affiliations:** 1 Institute of Pharmaceutical Science and Technology, College of Pharmacy, Hanyang University, 55 Hanyangdaehak-ro, Sangnok-gu, Ansan, Gyeonggi-do 15588, Korea; alam.zeb@riphah.edu.pk (A.Z.); bradchoi@hanyang.ac.kr (H.-I.C.); lch2162@hanyang.ac.kr (C.-H.L.); tjddnd55@hanyang.ac.kr (S.-W.B.); qleh0826@hanyang.ac.kr (C.-W.L.); onbae@hanyang.ac.kr (O.-N.B.); 2 Riphah Institute of Pharmaceutical Science, Riphah International University, Islamabad 44000, Pakistan; isra.rana@riphah.edu.pk (I.R.); namrah.khan@riphah.edu.pk (N.K.); tabassamsadia96@yahoo.com (S.T.A.); sahar.tariq2011@gmail.com (N.u.S.); aroojalvi@hotmail.com (A.M.A.); fawad.shah@riphah.edu.pk (F.A.S.); 3 Department of Pharmacy, Quaid-i-Azam University, Islamabad 45320, Pakistan; fudin@qau.edu.pk; 4 Institute of Drug Research and Development, College of Pharmacy, Chungnam National University, 99 Daehak-ro, Yuseong-gu, Daejeon 34134, Korea

**Keywords:** biopharmaceuticals, recombinant DNA technology, delivery and formulation challenges, nanocarriers, proteins, monoclonal antibodies, enzymes, vaccines, cytokines, hormones

## Abstract

During the past two decades, the clinical use of biopharmaceutical products has markedly increased because of their obvious advantages over conventional small-molecule drug products. These advantages include better specificity, potency, targeting abilities, and reduced side effects. Despite the substantial clinical and commercial success, the macromolecular structure and intrinsic instability of biopharmaceuticals make their formulation and administration challenging and render parenteral delivery as the only viable option in most cases. The use of nanocarriers for efficient delivery of biopharmaceuticals is essential due to their practical benefits such as protecting from degradation in a hostile physiological environment, enhancing plasma half-life and retention time, facilitating absorption through the epithelium, providing site-specific delivery, and improving access to intracellular targets. In the current review, we highlight the clinical and commercial success of biopharmaceuticals and the overall applications and potential of nanocarriers in biopharmaceuticals delivery. Effective applications of nanocarriers for biopharmaceuticals delivery via invasive and noninvasive routes (oral, pulmonary, nasal, and skin) are presented here. The presented data undoubtedly demonstrate the great potential of combining nanocarriers with biopharmaceuticals to improve healthcare products in the future clinical landscape. In conclusion, nanocarriers are promising delivery tool for the hormones, cytokines, nucleic acids, vaccines, antibodies, enzymes, and gene- and cell-based therapeutics for the treatment of multiple pathological conditions.

## 1. Introduction

Biopharmaceuticals (also called biologics) are therapeutic products derived from biological sources including microorganisms, plants and animals, and they are mostly produced using advanced biotechnologies such as genetic engineering or hybridoma technique [1]. The major classes of biopharmaceuticals are enzymes, vaccines, monoclonal antibodies (mAbs), cytokines, hormones, recombinant blood products, hematopoietic growth factors, nucleic acid-based products (DNA and RNA), and gene- and cell-based therapeutics [2]. Biopharmaceuticals have larger and more complex structures than conventional small-molecule drugs [3]. As biopharmaceuticals possess their own unique and promising features, they have been enormously investigated in the past two decades by researchers who have explored their therapeutic potential and worked to address their shortcomings. The advent of biopharmaceuticals has brought a radical change to the pharmaceutical industry by modernizing the treatment of numerous life-threatening ailments, including cancers, hematological problems, diabetes, and immune diseases, and by providing enhanced patient care and valuable targeted therapies [4].

Biopharmaceuticals offer better specificity, potency, and targeting ability than conventional therapeutic agents along with reduced side effects, shorter times for development and approval, and better patent protection [5]. The structural complexity and macromolecular nature of biopharmaceuticals contribute to their high specificity and potency but simultaneously pose challenges in formulation, delivery, and regulatory evaluation [6,7]. Other areas of concern for biopharmaceuticals are immunogenicity, heterogeneous nature, rapid clearance from systemic circulation, intrinsic instability, and limited permeability across biological barriers [8,9]. These concerns make biopharmaceuticals challenging molecules in development and reduce their formulation and delivery options. 

In recent years, nanotechnology has emerged as an efficient tool to circumvent the drawbacks of conventional drug delivery systems. Nanocarriers can modify the basic properties and bioactivity of their encapsulated moieties for improved pharmacokinetic and biodistribution profiles, reduced toxicity, controlled release, enhanced solubility and stability, and site-specific delivery of their payload [10,11]. Furthermore, nanocarriers can be made to have a wide range of physicochemical characteristics by altering their composition, shape, size, and surface properties [12,13]. Nanocarriers can generally be categorized into organic and inorganic systems. The organic nanocarriers include liposomes, lipid nanoparticles, polymeric nanoparticles, dendrimers, micelles, and virus-like particles (VLPs), whereas inorganic nanocarriers include mesoporous silica nanoparticles (MSNs) and metallic nanoparticles [14]. Liposomes are spherical vesicles consisting of an aqueous phase enclosed by lipid bilayers of natural or synthetic phospholipids and cholesterol. They may vary in their physical and chemical properties depending on the composition and method of preparation. Liposomes act as suitable carriers for biopharmaceutical delivery due to their safety, versatile characteristics, and easy surface modifications [15,16]. Lipid nanoparticles are composed of triglycerides, partial glycerides, fatty acids, and waxes along with different surfactant combinations. The particle size of lipid nanoparticles is generally below 1 µm and demonstrates efficient and targeted drug delivery [17,18]. In polymeric nanoparticles, biocompatible and nontoxic natural or synthetic polymers are utilized to synthesize nanosized carriers. They contain either vesicular (nanocapsules) or matrix (nanospheres) systems [19]. Polymeric micelles are self-assembled carriers of block copolymers and consist of core–shell structure. The particle size, shape, and critical micelle concentration of polymeric micelles could be controlled by the structural and physical properties of block copolymers [20]. Dendrimers are organic nanocarriers having branched structures originating from a central core. Drug molecules are attached to dendrimers in a capsule or complex form, and surface modification is possible through physical and chemical linkages [21]. Nanogels are submicron-sized three-dimensional networks formed by physical or chemical crosslinking of polymers. Nanogels are attractive nanocarriers due to excellent drug loading capacity, high stability, biologic consistence, and stimuli-responsiveness to ionic strength, pH, and temperature. In addition, the cross-linked networks allow nanogels to swell and absorb high amounts of water or biological fluids. These unique features make them promising drug delivery tool [22,23]. VLPs are self-assembled protein cages from different virus sources and have uniform nanostructures and well-defined geometry for drug delivery and imaging applications [24]. MSNs are organized as honeycomb-like structures with hundreds of pores containing drug molecules. The diameter of pores can be controlled in a range of 2–50 nm to allow the loading of large amount of drug [25]. Gold nanoparticles are composed of gold atoms functionalized with thiol groups. They are nontoxic to human cell lines and offer sufficient colloidal stability, high compatibility, low toxicity, and surface functionalization [26].

Nanocarriers have already shown their potential to eliminate the difficulties in delivering macromolecular therapeutics and are expected to make biopharmaceuticals more appealing in future clinical applications. In this review, we highlight the clinical and commercial success of biopharmaceuticals and then describe in detail (i) the major challenges to successful delivery of biopharmaceuticals, (ii) the application of nanocarriers to overcome those delivery and formulation challenges, and (iii) the hurdles in clinical translation of nanocarriers. 

## 2. Overview of the Clinical and Commercial Success of Biopharmaceuticals

The first biopharmaceutical product, human insulin, created using recombinant DNA technology received the US FDA approval and was launched in 1982 [5]. The first therapeutic mAb found its way to market with the FDA approval of muromonab-CD3 in 1986 for the treatment of acute transplant rejection [27]. Recombinant DNA and hybridoma technologies have revolutionized the pharmaceutical industry and have produced many blockbuster biopharmaceuticals. Within a few years, the development and marketing of recombinant proteins such as interferons (α, β, and γ) had greatly expanded the biopharmaceutical industry. A variety of promising technologies such as genome-based techniques, design of chemically modified cells, improved production of mAbs, effective cancer therapies, and enhanced vaccine development processes have made the biopharmaceutical industry a rapidly growing sector [28]. Biopharmaceuticals offer specific and targeted therapies for life-threatening disorders and are currently being produced on a large scale to cater the diverse unmet medical needs of patients.

**Figure 1:**
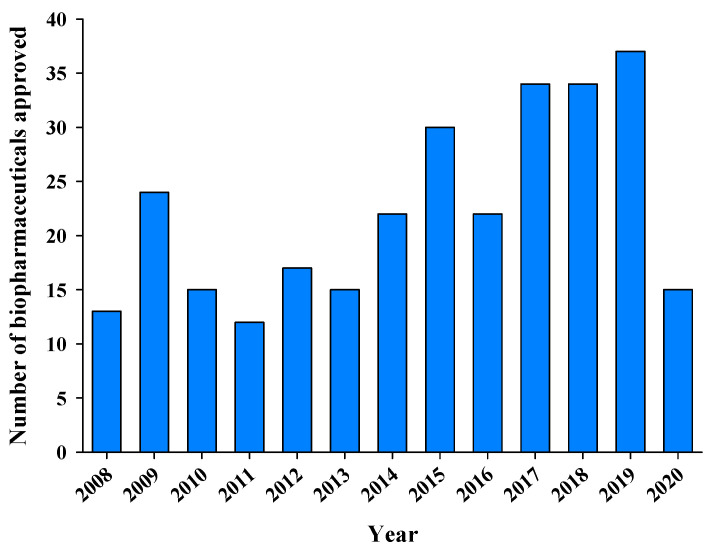
The number of biopharmaceuticals approved by the FDA from 2008 to 2020 (http://www.biopharma.com/approvals).

During the past two decades, the number of FDA approvals granted to biopharmaceuticals has increased substantially due to the development of efficient engineering processes, the discovery of new drug targets, and a better understanding of biopharmaceuticals fate in vivo [9]. The number of commercialized products will increase further with the arrival of generic versions when many approved biopharmaceuticals begin to come off-patent in the next few years. The major contributors to the clinical and commercial success of biopharmaceuticals are recombinant proteins and mAbs, which have provided major breakthroughs in oncology and the treatment of autoimmune disorders [29]. The worldwide sales revenue generated by biopharmaceuticals reached US$140 billion in 2013, with about half (~US$75 billion) of the total revenue contributed by mAbs [30]. Many biopharmaceuticals have achieved blockbuster status with individual annual revenue exceeding US$1 billion [29]. Furthermore, biopharmaceuticals are expected to account for more than 70% of new drug approvals by 2025 [7]. From 2008 to 2011, 64 biopharmaceuticals received FDA approval (Figure 1); the number increased to 84 over the next 4 years (2012–2015) and again to 127 in 2016–2019 [31]. A detailed description of the biopharmaceuticals approved in the past 3 years (2018–2020) is presented in Table 1 [31]. These statistics on its clinical and commercial success indicates the major impact of biopharmaceuticals on healthcare and their importance is expected to continue increasing.

## 3. Challenges in the Successful Delivery of Biopharmaceuticals

**Figure 2:**
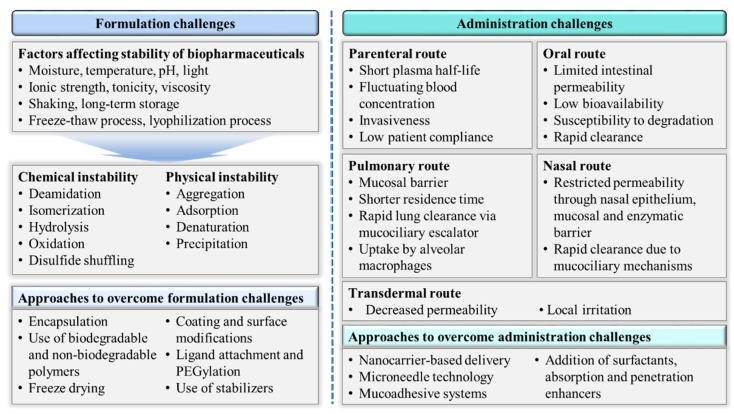
Formulation and administration challenges in delivering biopharmaceuticals.

The formulation and administration strategy for a particular drug is generally dictated by its inherent physicochemical and biological properties, and the adopted strategy has a major effect on the pharmacological performance of drug. In this regard, biopharmaceuticals are a unique class of therapeutics with a set of characteristics that differs distinctly from those found in traditional small-molecule drugs. The large and complex molecular structures of biopharmaceuticals, coupled with their intrinsic instability, create more challenges than success [32]. Those drawbacks have prompted researchers to design and develop new formulations that can deliver biopharmaceuticals efficiently. The inherent challenges in the formulation and administration of biopharmaceuticals are described in this section and summarized in Figure 2.

### 3.1. Formulation Challenges

Biopharmaceuticals, mostly protein-based products, present specific challenges in handling, formulation, storage, and transportation. Overcoming the inherent instability of biopharmaceuticals is one of the most important challenges. The therapeutic activity of biopharmaceuticals depends on a complicated three-dimensional shape that is based on secondary, tertiary, and, sometimes, quaternary structures. Any alteration in their conformational structure renders them not only inactive but also immunogenic [2,3]. Biopharmaceuticals are thus very delicate molecules whose conformational structures are easily altered by oxidation, hydrolysis, deamidation, isomerization, disulfide shuffling, adsorption, aggregation, denaturation, and precipitation [33]. These instabilities are triggered when biopharmaceuticals are exposed to extreme temperature or pH, high tonicity or osmolality, agitation, light, sheer forces, metals, and organic solvents [34]. The high viscosity of concentrated solutions is another area of concern for biopharmaceuticals because it makes them difficult to administer by injection. Formulation design is therefore geared to consider the ingredients, physical state, handling, and storage conditions of biopharmaceuticals to optimize their therapeutic outcomes and reduce adverse events [35]. 

### 3.2. Administration Challenges

The administration route of therapeutic intervention is an important factor that dictates its pharmacokinetics, biodistribution, and efficacy. Parenteral administration (intravenous, intramuscular, or subcutaneous injection) has been the primary and undoubtedly most suitable delivery mode for biopharmaceuticals because of their high molecular weight and physicochemical instability in the harsh environment encountered by other routes of administration [6]. However, parenteral administration has its own drawbacks such as invasiveness, short plasma half-life, frequent dosing, and fluctuating drug concentration in blood [36,37]. Furthermore, long-term and frequent injection is an important issue for patients who administer biopharmaceuticals to manage chronic diseases such as cancers and immunological disorders. To improve patient compliance and convenience, lots of formulation have been explored to deliver biopharmaceuticals via noninvasive routes (oral, transdermal, pulmonary, and nasal). Successful noninvasive delivery of biopharmaceuticals remains a challenge since each route presents its own distinct problems.

Oral administration remains the most preferred mode of noninvasive drug delivery for its convenience and acceptability to patients. However, the large molecular size, hydrophilicity, and inherent instability of biopharmaceuticals poses challenges such as limited intestinal permeability, low bioavailability, and susceptibility to degradation in the harsh gastrointestinal environment [13,32]. The high molecular weight (>3000 Da) and high hydrophilicity of biopharmaceuticals are the ultimate obstacle to successful oral administration because intestinal absorption via transcellular pathways is only feasible for lipophilic molecules with a molecular weight below 700 Da [38]. Paracellular route of absorption for hydrophilic molecules are also unavailable for macromolecular biopharmaceuticals owing to the tight junctions in intestinal epithelium [39]. In addition, the intestinal mucosal layer hinders the permeability of biopharmaceuticals across the epithelium through its barrier property and repulsive forces between the negatively charged biopharmaceuticals and the mucosal layer, which restrict their close contact and result in rapid clearance [40]. Another obstacle to the successful oral delivery of biopharmaceuticals is their propensity for proteolytic degradation in the gastrointestinal tract and denaturation in the acidic stomach environment [41,42]. The formulation approach has focused on combating these physical and biochemical barriers to protect biopharmaceuticals from the gastrointestinal environment and augment their oral bioavailability. 

Skin delivery of biopharmaceuticals is a convenient and noninvasive route of administration that addresses the major drawbacks of oral and parenteral delivery. However, the outermost layer of skin, the stratum corneum, has excellent barrier capabilities allowing this route to permeate only a few molecules with a specific set of physicochemical characteristics such as low molecular weight (<500 Da), a balance of lipophilicity (log P = 1–3) and water solubility (>1 mg/mL), a modest melting point (<200 °C) and a daily required dose in the range of a few milligrams [12,43]. Since most biopharmaceuticals are hydrophilic macromolecules, they do not possess these characteristics suitable for administration through skin. A variety of techniques has been used to alter the permeability of the stratum corneum and expand the number of biopharmaceuticals for transdermal delivery [44,45]. 

Pulmonary delivery is another noninvasive and easily accessible alternative to parenteral delivery that provides a large surface area, thin physical barrier, rich blood supply, fast systemic delivery, mild environment, and avoidance of first-pass metabolism [46]. Challenges to the pulmonary delivery of biopharmaceuticals include the restricted absorption due to large molecular size, hydrophilicity, and the barrier function of the mucosal layer that covers the epithelium in the airways. In addition, the short residence time of biopharmaceuticals is resulted from rapid lung clearance via the mucociliary escalator and uptake by alveolar macrophages [47]. Another limitation of developing aerosol formulation for pulmonary delivery is the additional requirement of special excipients such as propellants, anti-foaming agents, metered valves, and special containers, thereby adding more cost to the final formulation [48]. Formulations intended for pulmonary delivery need to be optimized in terms of particle size, size distribution, surface properties, release rate, and dose. Furthermore, the physiochemical characteristics of inhaled therapeutics such as their physical state, molecular weight, charge, solubility, hydrophilicity, and lipophilicity must be considered when designing biopharmaceuticals formulation for pulmonary delivery [49].

Nasal route offers a porous epithelium and a highly vascularized large surface area, and thereby leads to rapid and systemic absorption of drugs [50]. However, the nasal administration of biopharmaceuticals has several limitations including restricted permeability of large molecules through the nasal epithelium, mucosal, and enzymatic barriers, and rapid clearance through mucociliary mechanisms [51]. Other noninvasive routes such as buccal, vaginal, rectal, and sublingual routes have also been investigated and shown potential for biopharmaceutical delivery, but they suffer from challenges similar to those faced by the aforementioned routes.

**Figure 3:**
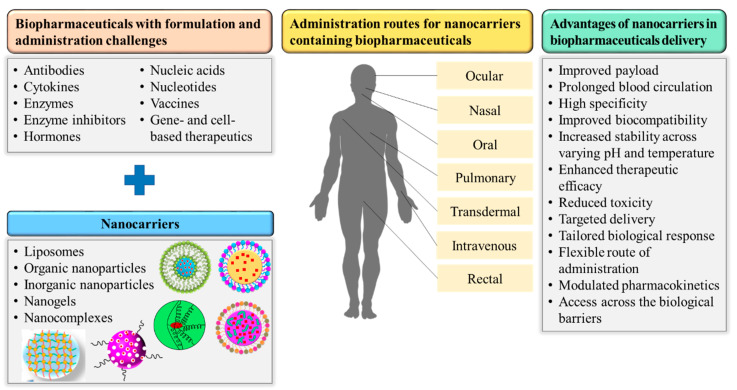
Nanocarrier-based approaches for efficient biopharmaceutical delivery.

Many of the formulation and administration challenges just discussed can be addressed by designing appropriate biodegradable and biocompatible nanoplatforms, which will improve not only therapeutic performance but also medical applications and clinical success [52,53,54]. The effective use of nanocarriers to deliver biopharmaceuticals for diagnostic, preventive, and therapeutic purposes has revolutionized the treatment of life-threatening diseases [55]. Nanocarriers have successfully addressed many of the drawbacks of conventional delivery systems including their non-specificity, adverse effects, and burst release. The successful use of nanotechnology in biopharmaceutical delivery will enhance patient acceptability and allow biologics to further dominate the drug market in the future. The application of various nanocarriers to address unmet needs in the formulation and administration of biopharmaceuticals is presented in the next section and depicted in Figure 3.

## 4. Applications of Nanocarriers in Successful Biopharmaceutical Delivery

**Figure 4:**
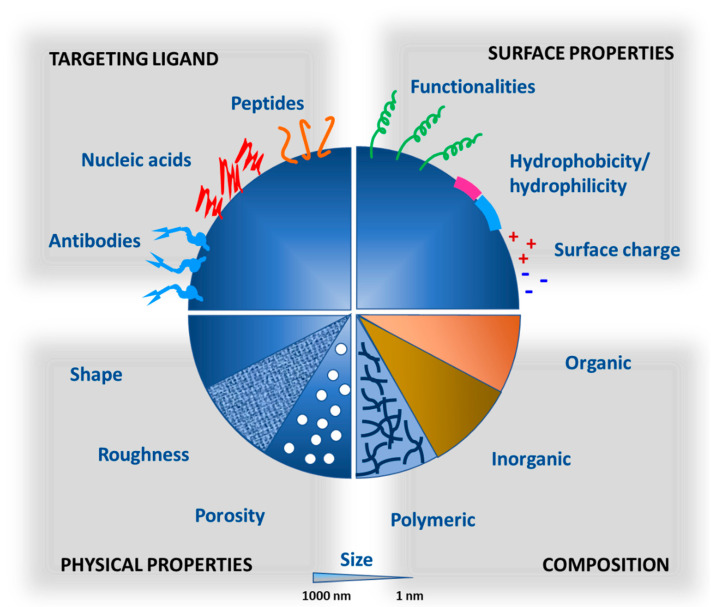
Biological and physicochemical properties of nanocarriers in modulating biopharmaceutical delivery.

Nanotechnology has been used in medicine for more than three decades and had tremendous success in effectively delivering bioactive molecules to a variety of inaccessible targets. The launch of successful nanocarrier-based formulations for small-molecule drugs such as Doxil^®^, DaunoXome^®^, Abraxane^®^, Onco TCS^®^, and Ambisome^®^ has opened windows for the exploration of nanotechnology to deliver macromolecular biopharmaceuticals [56]. ONPATTRO^®^ was the first FDA approved RNAi product, formulated as a lipid complex, for the treatment of polyneuropathy in hereditary transthyretin-mediated amyloidosis. Nanocarriers augment the therapeutic outcomes of biopharmaceuticals by protecting them from degradation in hostile biological environments, enhancing their half-life and retention time in blood, facilitating absorption through epithelium, providing control over drug release and site-targeted delivery, and improving access to intracellular targets [52,57]. Nanocarriers can be fabricated using organic or inorganic materials, and their physicochemical and biological properties such as particle size, shape, porosity, charge, and surface chemistry could be tuned. The composition, physical and surface properties, and functionalization of nanocarriers dictate their biological behavior and ultimately the therapeutic efficiency of the loaded bioactive molecules (Figure 4). The particle size, surface area, and charge of nanoparticles are associated with increased solubility, stability, oral absorption, and their ability to reach the target site [58,59]. Surface modification of nanocarriers with hydrophilic polymers (e.g., polyethylene glycol (PEG)) prolongs their systemic circulation [60]. Similarly, functionalization of nanocarriers with targeting ligand such as antibody and peptide enhances their selectivity to a specific target including the brain and tumor [61]. Nanocarrier-based formulations of biopharmaceuticals are expected to hit the market in the near future while keeping in view the current explosive growth and interest in this field [62]. Although the compositional and structural features of various nanocarriers have been reviewed previously [14], their applications in the effective delivery of major biopharmaceuticals are newly presented here (Table 2).

### 4.1. Nanocarriers-Mediated Hormones Delivery

Hormones are the most explored biopharmaceuticals because of their clinical applications in highly prevalent diseases. Therapeutic hormones have been encapsulated in nanocarriers for efficient delivery across physiochemical and biological barriers via different routes. Insulin is a representative example, and most studies aim to improve its bioavailability by finding more effective routes than subcutaneous injection. Submicron solid lipid nanoparticles (SLNs) have shown potential to protect encapsulated peptides from degradation in the gastrointestinal tract and promote transmucosal delivery via different mechanisms including mucoadhesion, internalization, and absorption enhancement [63,64]. Lectin-modified SLNs were developed to enhance the oral bioavailability of insulin in a rat model [65], and they improved in vitro stability of insulin against degradation by acidic pH and proteolytic enzymes. In addition, lectin-modified SLNs demonstrated that the bioavailability after oral administration was 7.11% higher than that after subcutaneous injection, indicating the facilitation of oral absorption by encapsulating insulin in SLNs. 

Polymeric nanoparticles have also been widely explored for efficient hormone delivery. For example, chitosan-coated nanoparticles were developed for oral administration of insulin. The prepared nanoparticles increased the paracellular permeability in Caco-2 cells and improved insulin stability during storage. Moreover, oral administration of the insulin-loaded nanoparticles decreased the blood glucose level in diabetic rats for 10 h via sustained release and absorption enhancement [66]. In another study, insulin-loaded nanoparticles were prepared with biodegradable polymer polylactic-co-glycolic acid (PLGA) and Eudragit^®^ RS to increase the penetration of insulin into the intestinal mucosa. The insulin-loaded PLGA/Eudragit^®^ RS nanoparticles showed high encapsulation efficiency (73.9%) with an average particle size of 285 nm and a zeta potential of +42 mV. The cationic PLGA/Eudragit^®^ RS nanoparticles were enclosed in enteric-coated capsules composed of hydroxypropyl methylcellulose phthalate (HP55) and showed promising in vivo antidiabetic activity for a prolonged period after oral administration [67]. The enteric coating with HP55 acted as a pH-sensitive barrier to retard insulin release in gastric fluid. Similarly, PLGA-based insulin nanoparticles embedded in a polyvinyl alcohol (PVA) hydrogel showed a sustained release rate that delivered the total amount of insulin over 24 h [68]. When folate-decorated PEGylated PLGA nanoparticles were orally administered, the bioavailability of insulin was doubled compared to subcutaneous injection without causing any hypoglycemic shock [69].

Colloidal nanotechnologies have also shown promising results in the delivery of many other hormones. Antiandrogen-loaded gold nanoparticles were prepared with thiol PEGylated antiandrogen and thiol polyethylene glycol stabilizer. The prepared nanoparticles had an optimal particle size (29 ± 4 nm) to achieve cellular internalization and accumulation at the tumor site. The PEGylation of gold nanoparticles provided steric stabilization in physiological media to escape immunogenic responses. The resulting nanoparticles that target GPRC6A and specifically antagonize the androgen receptor have reduced cell proliferation and proven to be a selective and potent treatment against hormone-insensitive and chemotherapy-resistant prostate cancer [70]. Peptide hormones such as human growth hormone (hGH), calcitonin, and melatonin suffer from aggregation, precipitation, and inactivation when exposed to varying pH, temperature, and ionic strength. These problems were mostly alleviated by formulating the peptides in pH-responsive, pH-dependent, or thermosensitive nanocarriers and by chemically stabilizing the hormones through PEGylation. The short plasma half-life of hGH requires frequent intravenous administration, leading to poor outcomes, reduced patient compliance, and increased toxicity. When hGH was incorporated into dual ionic thermosensitive nanogels for sustained delivery, the initial burst release was reduced and better in vitro and in vivo correlation was found [71]. The nanogels had a particle size of 500 nm and a zeta potential of +8 mV and demonstrated a 13-fold increase in AUC and enhanced bioavailability compared with hGH solution in a hypophysectomized rat model. 

Calcitonin is a peptide hormone that regulates calcium homeostasis and rapidly lowers circulating calcium levels by inhibiting calcium efflux from bone. Calcitonin has been clinically used for the treatment of osteoporosis as it prevents bone resorption [72]. The poor oral bioavailability of calcitonin (<0.1%) is due to active proteolytic degradation in the gut. Chitosan-modified PLGA nanoparticles containing salmon calcitonin were prepared using emulsification technique to overcome its poor oral bioavailability. The prepared spherical nanoparticles (430–590 nm) showed high encapsulation efficiency and improved hypocalcemic effects of calcitonin via improved oral absorption and sustained release [73]. Similarly, hydrogel-based nanoparticles prepared with a thiomer derivatives of glycol chitosan and thioglycolic acid significantly improved the pulmonary delivery of calcitonin. Reportedly, the nanoparticles (200–300 nm), which were prepared using an ionic gelation method and had a net positive surface charge, showed high calcitonin encapsulation and a pronounced hypocalcemic effect for up to 24 h [74]. 

Melatonin is an endogenous bioactive substance that regulates body temperature and endocrine, immune, and nervous systems. Despite its rapid dissolution, melatonin shows a very low bioavailability of only ~15%. Melatonin-loaded nanoparticles were prepared with gelatin, polylactic acid, and chitosan, and evaluated for their effects on depressive behaviors and hormone secretion in pinealectomized rats. The melatonin-loaded nanoparticles demonstrated controlled release profiles at various pHs and improved antidepressant activity and blunt negative feedback along the hypothalamic–pituitary–adrenal (HPA) axis compared with free melatonin [75]. Estrogens are endogenous substances involved in the growth and maintenance of the female reproductive system and sexual characteristics. Estradiol is a principal and potent estrogen used for preventing postmenopausal osteoporosis, managing menopausal symptoms, providing hormone replacement therapy and reducing the incidence of mammary cancers [76,77]. The low bioavailability and extensive hepatic metabolism of estradiol creates a need for frequent dosing that causes various side effects. To enhance its oral bioavailability, PLGA nanoparticles of estradiol were prepared using PVA or didodecyldimethylammonium bromide as a stabilizer. The resulting nanoparticles had a particle size of 410 ± 39.4 and 148 ± 10.7 nm and showed sustained release for 45 and 31 days, respectively. In addition, intestinal uptake, histopathological analyses, and blood counts indicated the effective delivery of estradiol via nanoparticles [78]. Similarly, estradiol-loaded PLGA nanoparticles administered via the skin were assessed for their ability to treat osteoporosis. The nanoparticles, which were prepared by solvent evaporation method, had a particle size of 153.3 ± 49.1 nm and encapsulation efficiency of 70.49 ± 3.94%. Enhanced in vivo skin permeation was verified when the nanoparticles were combined with iontophoresis [79]. 

### 4.2. Nanocarriers-Mediated Cytokines Delivery

Cytokines such as interleukins (ILs), interferons (IFNs), and tumor necrosis factors (TNFs) are essential modulators in maintaining immune homeostasis and inflammatory responses, combating pathogens and enforcing tolerogenic mechanisms [80]. Cytokines produced through recombinant DNA technology are generally administered to modulate immune responses to cancer, autoimmune disorders, or infectious diseases, and their adjuvant properties can increase vaccine efficacy. Despite the therapeutic potential of cytokines, multiple problems associated with the effective delivery limit their efficacy. Intravenously administered cytokines are usually inactivated by protein degradation or binding to nonspecific receptors. The repeated administration of cytokines leads to increased systemic circulation, which can eventually produce a toxic dose. To address these challenges, various polymeric and lipid-based nanocarriers for cytokine delivery have been investigated. 

Granulocyte-macrophage colony-stimulating factor (GM-CSF) and granulocyte colony-stimulating factor (G-CSF) were encapsulated in dextran nanoparticles with a size of 200–500 nm and a high entrapment efficiency (>98%). The nanoparticles preserved the bioactivity of delicate proteins, preventing their aggregation and ensuring their stability in an acidic environment [81]. In another study, a stable oil-in-water nanoemulsion was prepared to effectively deliver IFN-γ and assessed for phagocytic activity and cytotoxicity in MCF-7 human breast cancer cells. The nanoemulsion was prepared using an ultrahomogenization technique with tricaprin, sorbitan oleate, polysorbate 80, and 1-butanol. The prepared nanoemulsion reduced the cell viability of MCF-7 cells without affecting the cell viability of phagocytes. In addition, the cellular activity of phagocytes was induced by the nanoemulsion as indicated by increased intracellular Ca^2+^ release in phagocytic cells. These results demonstrated the potential of an IFN-γ-loaded nanoemulsion to modulate the immune response and produce anticancer activity [82]. IFN-β-1a has been used to combat autoimmune diseases such as multiple sclerosis. It was reported that IFN-β-1a-loaded PLGA and PEG-PLGA nanoparticles sustained the in vitro release of IFN-β-1a and diminished cytokine toxicity in hepatocytes [83]. Despite the excellent clinical efficacy of IFN-α in treating cancers and viral infections, its use is limited by its high parenteral dose and side effects. IFN-α-loaded chitosan nanoparticles were developed for oral delivery by ionotropic gelation and exhibited a particle size of 36 ± 8 nm and 100% encapsulation efficiency. Within 1 h after oral administration, the chitosan nanoparticles produced the detectable plasma levels of IFN-α [84]. 

Regulatory T cells (Treg) play an essential role in maintaining the tumor microenvironment and thereby suppressing immunotherapy. Effective strategies are needed to modulate the tumorigenic effects of these cells. Liposomes conjugated with Treg cells were explored for their ability to effectively deliver cytokines to a tumor site. Based on the chemotaxis of tumor microenvironment, pH-responsive Treg-loaded liposomes were guided toward the acidic tumor environment to produce efficient tumor suppression in situ and augment cancer immunotherapy [85].

Mesoporous silica nanoparticles (MSNs) with extralarge pores were prepared for in vivo IL-4 cytokine delivery. The IL-4-loaded MSNs targeted phagocytic myeloid cells such as neutrophils, monocytes, macrophages, and dendritic cells, and also elicited in vivo M2 macrophage polarization to modulate immune systems through the targeted delivery of cytokines [86]. Adoptive cell therapy (ACT) isolates autologous tumor-specific T cells from a cancer patient followed by ex vivo activation and enhancement, and then the cells are infused back into the individual to eliminate metastatic tumors. The major limitation of ACT therapy is the rapid loss of effector T cells in the highly immunosuppressive tumor microenvironment. PEGylated liposomes have been tested to deliver IL-2 to T cells in vivo since supporting cytokines are required to enhance the efficacy of T cell therapy. The liposomes were reported to target ACT cells and enhance T cell proliferation in the tumor microenvironment [87]. 

### 4.3. Nanocarriers-Mediated Nucleic Acid and Nucleotide Delivery

Nucleotide delivery is one of the biggest challenges of nucleic acid-based biopharmaceuticals because of its large molecular size, negative charge, hydrophilicity, and degradation by nuclease [88]. The effective delivery of such molecules using colloidal nanotechnology has been widely investigated. Small interfering RNA (siRNA) have emerged as a promising therapeutic against a variety of pathological conditions including viral infections, tumors, genetic disorders, and autoimmune diseases [89]. However, the inherent problems of free siRNA are limited ability to pass through cell membranes, half-life of less than 1 h, and instability in blood [90]. Carrier systems are required to deliver these nucleotides to the targeted site and overcome the associated limitations. siRNA-loaded polymeric nanoparticles were prepared using PVA modified with diamine moieties and PLGA (DEAPA-PVA-g-PLGA) and evaluated for their cellular uptake and the intracellular localization [91]. The resulting nanoparticles showed high and rapid cellular uptake and localization in endosomes and lysosomes, demonstrating efficient delivery of siRNA for gene silencing. 

Cytokines and chemokines play an important role in the progression of inflammatory bowel disease and systemic neutralization by antibodies has also been reported in some patients. Using siRNA to target cytokine signaling could be a useful therapeutic strategy for the treatment of colonic inflammation. Calcium phosphate-PLGA-PEI multishell nanoparticles exhibited rapid cellular uptake, significant in vitro gene silencing and negligible toxicity resulting in a remarkable decrease in the target genes evidenced by colonic biopsies [92]. The potential of CD98 siRNA-loaded nanoparticles to reduce CD98 expression and treat nonalcoholic fatty liver disease was investigated [93]. Double emulsion solvent evaporation technique was used to synthesize CD98 siRNA-loaded nanoparticles with a size of 275 nm. These nanoparticles significantly downregulated the expression of CD98 in HepG2 cells, along with a reduction in liver alanine aminotransferase (ALT) in blood. 

To deliver CD73-specific siRNA, chitosan lactate nanoparticles were prepared and found to cause potent inhibition of tumor cell proliferation, a reduction in angiogenesis, and downregulation of angiogenesis-promoting factors. Moreover, an analysis of leukocytes derived from tumor samples determined a lower ability to secrete angiogenesis-promoting factors following CD73 silencing, which led to tumor suppression [94]. Natural polysaccharide chitosan nanoparticles containing a nucleotide and its analogue were investigated for efficient, specific, and targeted in vitro delivery of the nucleotide to the cell cytoplasm [95]. The antiapoptotic gene bcl-2 is overexpressed and frequently evident in different tumors. G3139 is an antisense oligonucleotide responsible for silencing Bcl-2 but has shown limited clinical efficacy. A G3139 oligonucleotide was prepared using a similar technique for gapmers and incorporated into lipid nanoparticles composed of 1,2-dioleoyl-3-trimethylammonium-propane, Tween 80, egg L-α-phosphatidylcholine, and cholesterol. The optimized nanoparticles had a particle size of 134 nm with efficient encapsulation and demonstrated a significant downregulation of the bcl-2 gene. Tumor proliferation and survival were also significantly reduced [96]. 

The nanocarriers-mediated delivery of RNA has also been investigated in nonhuman primates. It was demonstrated that self-amplifying mRNA delivered via nanoemulsion complex elicited an excellent immune response in nonhuman primates comparable to a viral delivery technology. The antibody and T cell responses were induced in nonhuman primates at relatively low doses [97]. Similarly, siRNA delivered as lipid-like material showed sufficient gene silencing in nonhuman primates after low-dose injection for hepatic delivery [98]. Lipoid-siRNA formulation showed a highly specific and targeted delivery to hepatic tissues with ~90% distribution in nonhuman primates. The in vivo efficacy was varied by changing formulation parameters such as particle size, nature of PEGylation and degree of drug loading [99]. Ionizable low-molecular weight polymeric nanoparticles demonstrated successful endothelial siRNA delivery and gene silencing in multiple nonhuman primates after systemic administration [100].

Codelivery of cytotoxic therapeutics in a single nanocarrier has also been widely investigated. Trilysinoyl oleylamide-based liposomes were prepared for codelivery of siRNA and an anticancer drug, suberoylanilide hydroxamic acid. Tumor growth was significantly reduced after intravenous administration in animal models. The siRNA incorporated in cationic liposomes silenced target genes both in vitro and in vivo [101]. Similarly, folate-modified multifunctional nanoassembly was investigated for the codelivery of iSur-pDNA and docetaxel in hepatocellular carcinoma. The nanocarriers showed particle size of around 200 nm with high encapsulation efficiency (~90%). Codelivery sufficiently increased cytotoxic effect of docetaxel in mouse hepatocellular carcinoma model [102]. Multiple gene silencing via a dual-gene targeted siRNA was explored for synergistic effects in cancer therapy. Two different sequences of siRNA were chemically combined into a single siRNA backbone and incorporated into chitosan nanoparticles. The nanoparticle-mediated codelivery of siRNA targeting VEGF and Bcl-2 showed sufficient dual gene silencing in tumor cells [103]. In another study, layer-by-layer nanoparticles were developed for codelivery of siRNA and doxorubicin to treat triple-negative breast cancer. The nanoparticles exhibited reduced gene expression in tumor cells up to 80% and potentiated doxorubicin-based chemotherapy in resistant cancers [104].

### 4.4. Nanocarriers-Mediated Vaccines Delivery

Vaccination is necessary to control infectious diseases, but vaccines against various infections face difficulties such as an inability to evoke a sufficient immune response, instability in biological environments, limited ability to penetrate biologic membranes, and hindrance in reaching the targeted site [105]. Nanoscale particles (i.e., smaller than 1000 nm) have been suggested to stabilize vaccines and could also act as adjuvants in their delivery [106]. Not only traditional vaccines, such as live attenuated microbes, killed microbes or components of microbes but also isolated proteins, polysaccharides, and naked DNA encapsulating the antigen are all being exploited in the preparation of vaccines [107]. In addition, self-replicating single-stranded RNA viruses have also been utilized as vectors for vaccine development. These replicon RNA vaccines have produced strong immune responses and generated sufficient neutralizing antibodies in animal models [108]. It is necessary to properly utilize the well-defined mechanisms of nanocarriers to deliver the vaccines to targeted cells. The immune response and potency of vaccine are largely influenced by physicochemical properties such as composition, particle size, particle shape, surface charge, and hydrophobicity [106].

To mediate viral clearance in hepatitis B infections, therapeutic vaccines capable of inducing T helper type 1 cells have been suggested. The therapeutic hepatitis B vaccine was formulated by encapsulating a viral core antigen (HBcAg) in PLGA nanoparticles with or without the aid of an immunomodulator (monophospholipid A). The prepared nanoparticles had a spherical shape, an average diameter of 300 nm and an encapsulation efficiency of 50%. The codelivery of HBcAg and monophospholipid A in a single immunization generated an increase in IFN-γ production in murine models, which led to an elevated immune response in the form of T helper type 1 cells [109]. The outbreak of Ebola virus disease in West Africa led to approximately 11,000 deaths and was marked as an endemic. There was an urgent need to develop an Ebola virus vaccine. Synthetic nanoparticles were suggested for use as a highly specific and immunogenic platform for delivering the Ebola virus vaccine. A recombinant viral antigen for the Ebola virus was incorporated in lipid-based nanoparticles called interbilayer-cross-linked multilamellar vesicles. The nanoparticles presented the efficient generation of germinal center B cells and induced an immune response by neutralizing antibodies [110].

The degradation of vaccines in the acidic gastric environment is another limitation to the effective oral delivery. PLGA-based nanoparticles were developed to encapsulate *Helicobacter pylori (H. pylori)* recombinant antigen for oral vaccination. A protective approach was used to prevent the development of *H. pylori* infections in animal models. It was demonstrated that the immunization with nanoparticles in mice induced the production of antibodies and memory T cells, and 43% of the mice were protected when subsequently infected with *H. pylori* [111]. Among bacterial pathogens, *Bacillus anthracis* and *Yersinia pestis*, which, respectively, causes anthrax and plague, are particularly lethal. A dual nanoparticle vaccine against anthrax and plague was formulated using bacteriophage T4 as a nanoplatform. The capsid of the phage T4 was conjugated with protective, capsular, and calcium-response V bacterial antigens. The nanoparticles produced an efficient immune response in mice, rats, and rabbits, and also displayed a sufficient protective effect when challenged with a toxic dose of both organisms, suggesting that phage T4 could be a unique platform for the delivery of vaccines [112].

Phage T4 has also been investigated to deliver viral vaccines. Human immunodeficiency virus (HIV) is the causative organism of acquired immunodeficiency syndrome. Although antiretroviral therapies have markedly reduced mortality from HIV, the efficacy of vaccines remains questionable. The inability to elicit an immune response, the production of weak neutralizing antibodies and the negligible protective response are some of the problems associated with viral vaccines [113]. Virus-like particles (VLPs) enveloping the gp140 glycoprotein were assessed for immunogenicity in a murine model after expression of HIV Env gp140 or gp41 glycoproteins in insect cells. From a neutralization assay, the VLPs produced an effective antibody response in animal models suggesting the possibility of a broad spectrum of viral epitopes that could be targeted by an immune response [114]. 

Messenger RNA (mRNA)-based vaccine is a novel approach to vaccine development that does not require integration into the host genome and potentially activates the cytotoxic immune system. However, the limited ability to enter antigen-presenting cells and high nuclease activity hinder the delivery of mRNA-based vaccines. The potential of cationic lipid-based nanoparticles as carriers for mRNA vaccines was investigated. The maturation of dendritic cells was increased by the use of mRNA vaccine–loaded nanocarriers with enhanced in vivo and in vitro stimulation and proliferation of antigen-specific T cells. The T cell response additionally decreased tumor activity in a lymphoma model [115]. Polylactic acid (PLA) nanoparticles were modified to deliver an mRNA vaccine to dendritic cells, which are known to induce the efficient cytotoxic activity in infections such as HIV and to attack tumors by stimulating both innate and adaptive immunity. The PLA nanoparticle-mediated delivery of an mRNA vaccine produced efficient uptake of the nanoparticles by dendritic cells through phagocytosis and clathrin-dependent endocytosis. It also modulated the immune response by activating endosomes and induced the expression of proteins and markers for adaptive immunity in vitro [116].

### 4.5. Nanocarriers-Mediated Antibodies Delivery

Therapeutic mAbs are intended for targeted delivery to the proteins responsible for the pathological condition and require high specificity to optimize therapeutic outcomes. Recombinant technologies allow the preparation and use of antibody fragments and mAbs with different sizes and effector functions [117]. Several mAbs are currently used in clinical practice to treat solid tumors, hematological cancers, inflammatory conditions, and various infections. Despite their wide range of therapeutic roles, mAbs face multiple barriers to therapeutic competence. Commercialized mAbs are known to circulate systemically rather than being deposited in the targeted tissues, and they thus require high dosing to achieve the required bioavailability. The relatively large size and hydrophilicity of mAbs also limit their penetrative capability, which affects their tissue distribution [118]. Biocompatible nanocarriers could improve antibody therapy by offering tailored properties and enhanced target specificity. 

Epidermal growth factor receptor (EGFR) plays a substantial role in the invasion and proliferation of cancer cells and modifies angiogenesis and apoptosis. PEG immunomicelles were developed to transport anti-EGFR antibodies to a target site, along with doxorubicin and superparamagnetic iron oxide. The nanosized micelles demonstrated high internalization of the anti-EGFR antibody in the A431 tumor cells, and the use of doxorubicin with the antibody produced extensive cytotoxicity in an in vitro analysis in EGFR-overexpressing cell lines [119]. Infliximab-loaded liposomes were reported to treat experimental autoimmune uveoretinitis. The nanosized liposomes demonstrated reduced ocular inflammation following intravitreal injection, without causing any toxicity [120]. Similarly, gastrointestinal inflammation was targeted using infliximab-loaded PEGylated polyester urethane nanoparticles. High cellular interaction and increased permeability through Caco-2 cell monolayers was observed, and the cytokine levels in inflamed monocytes were reduced [121]. Nanocomplexes of *N*,*N*,*N*-trimethyl chitosan chloride were prepared by ionic gelation and loaded with an antibody against human liver heparan sulfate proteoglycan to target hepatocellular carcinoma. These nanocomplexes were investigated for their uptake by mouse monocyte models of cancer and demonstrated high internalization, greater cytotoxicity, and an increased half-life of the antibodies compared with the antibody treatment alone [122]. 

Apart from loading mAbs within a nanocarrier, surface functionalization of nanoparticles with antibodies increases targeting and specificity, thereby enables better therapeutic outcomes. The chemical conjugation of antibodies on a nanocarrier surface usually produces high specificity and increased cytotoxicity in cancer cells. Subsequent drug internalization can also be enhanced using PEGylated nanoparticles that incorporate the drug. In intrinsic drug-resistant breast cancer, the chemical conjugation of anti-human epidermal growth factor receptor 2 (HER2) antibodies on PEGylated liposomal doxorubicin proved to be effective [123]. The humanized bispecific antibody showed sufficient affinity with mPEG and up to 200-fold increased cytotoxicity in cells overexpressing HER2. The accumulation of doxorubicin in cancerous cells of tumor-bearing mice was also improved by the treatment, suggesting the therapeutic efficacy of PEGylated liposomal doxorubicin. 

Human serum albumin (HSA) nanoparticles are also used to actively target various tumor cells because of their superficial functional groups. HER2 is significantly expressed in various tumors, making it a potential target for therapeutic mAbs. For example, a novel mAb (IF2) was conjugated on the surface of an HSA nanocarrier and targeted against HER2 receptors. High internalization and sufficient cytotoxicity on the surface of BT474 cells was achieved in vitro by the PEGylated HSA nanocarrier tagged with IF2 [124]. Cetuximab-conjugated PLGA nanoparticles carrying paclitaxel were also investigated to target EGFR in nonsmall cell lung carcinoma [125], and sufficient internalization and cellular cytotoxicity were observed. In addition, high tolerability and enhanced efficacy were demonstrated in a metastatic lung cancer model, along with high tumor inhibition and an increased survival rate following intravenous administration in mice. 

To enhance effective targeting and cytotoxicity in ovarian cancer, transferrin and mAb 2C5-modified dual ligand-targeted PEG-phosphatidylethanolamine micelles showed increased cellular internalization compared with plain and single ligand-targeted micelles via endocytosis in tumor cells [126]. Similarly, gold nanoparticles bioconjugated with cetuximab to target EGFR were investigated in cell lines overexpressing EGFR and showed consistent and effective targeting both in vitro and in vivo in NMRI nude mice bearing A431 epidermoid carcinoma tumors [127]. Methotrexate HSA nanoparticles with a surface conjugation of trastuzumab molecules were investigated for their cytotoxic potential against HER2 cells and showed effective binding, internalization, and cytotoxicity, and they increased the therapeutic efficacy of the methotrexate [128]. Antibody-tagged nanocarriers also effectively deliver cytotoxic drugs to tumor sites without inflicting side effects. Arsenic trioxide has high potential in targeting solid tumors, but it possesses the drawback of affecting healthy cells. Therefore, an amphiphilic diblock copolymer of PEG and poly(d, l-lactide) was used to prepare nanocarriers encapsulates with arsenite ion. Surface functionalization with an anti-CD44v6 antibody allowed successful targeting of the CD44v6 receptors overexpressed in various cancers, such as hepatic, pancreatic, gastric, and colorectal. The consequent delivery of a cytotoxic drug via the antibody-conjugated nanocarrier had high therapeutic efficacy and targeted tumor specificity, resulting in the provision of a safe platform for anticancer drugs that reduced side effects [129].

Brain delivery of a centrally acting drug loaded in a nanocarrier is also facilitated by conjugating antibodies on the surface of the nanocarriers. A Fas ligand antibody tagged on a PEGylated nanocarrier demonstrated effective penetration through the blood–brain barriers (BBB), along with selective targeting and adequate therapeutic efficacy in the ischemic brain regions [130]. Similarly, surface functionalization of peptide iAβ5-loaded PLGA nanoparticles with antitransferrin and antiamyloid antibodies demonstrated high permeability through the BBB, as evaluated using porcine brain capillary endothelial cells. These nanoparticles also demonstrated sustained drug release and good therapeutic outcomes in Alzheimer’s disease [131]. Targeting brain tumors, such as glioblastoma, is another challenge in drug delivery. Cisplatin-loaded nanogels modified with antibodies against the membrane protein connexin 43 and brain-specific anion transporter were investigated for treating intracranial gliomas [132]. Following the administration of the conjugated nanogels, the tumor volume in mice was reduced and the survival rate was significantly increased. 

### 4.6. Nanocarriers-Mediated Delivery of Enzymes and Enzyme Inhibitors

The deficiency of the enzyme α-galactosidase results in the development of Fabry disease, a rare X-linked disorder of lysosomal storage. The only treatment currently available is recombinant α-galactosidase. However, ensuring the maximum delivery and an effective concentration of enzyme at the targeted site is difficult. HSA and 30Kc19 protein nanoparticles were investigated to address the problems associated with enzyme replacement therapy [133]. Enhancement of α-galactosidase activity and stability, along with minimal toxicity was observed by incorporating α-galactosidase in the nanocarriers. Gaucher’s disease is a common lysosomal disorder that involves a deficiency in β-galactosidase and it was the first lysosomal disease to be treated with enzyme replacement therapy. PLA nanoparticles with a surface coating of chitosan were studied for mucosal delivery of β-galactosidase. A solvent diffusion technique produced stable nanocarriers with sufficient tolerance against proteolytic and hydrolytic activity. Following oral administration, an increase in the half-life of the enzyme was also observed [134]. Similarly, cysteine proteinase type-I incorporated in SLNs was investigated in C57BL/6 mice to treat Leishmania major infection [135]. The nanoparticles produced a strong antigen-specific T-helper type 1 immune response that decreased the parasite burden, as assessed through lymph node cells. Moreover, the immune response inflicted by cytokines was also increased. 

Thromboembolic diseases also require enzyme treatment, specifically plasminogen activators. Excessive inactivation, clearance, short half-life, bleeding complications, and nonspecific tissue targeting are some of the problems associated with the therapy. Nanocarriers are used to avoid these drawbacks and to produce the desired outcomes. Liposomes loaded with tissue plasminogen activator (tPA) were investigated following subconjunctival injection in rabbit eyes. The absorption rates in subconjunctival hemorrhages were greatly affected by the liposomes, and the activity of the tPA was significantly prolonged [136]. In another study, the thrombolytic potential of tPA was evaluated by loading it into liposomes. Better molecular targeting and the low dose requirement of the tPA-liposomes add to their merits as an alternative to tPA alone. Moreover, the fibrin-targeting ability of the liposomal formulation enabled it to be used as an effective preparation against ischemic strokes [137]. Likewise, streptokinase and chitosan nanoparticles were prepared and evaluated for their thrombolytic activity [138]. The nanoparticles thus prepared showed a slight toxic effect on human fetal lung fibroblast cells (Mrc-5), as evaluated by MTT and euglobulin clot lysis assays. RGD-conjugated liposomes were studied in another investigation to determine their biodistribution and thrombolytic activity. The conjugated liposomes were efficiently delivered to the site of a blood clot in a rat’s carotid artery and demonstrated high thrombolytic activity [139]. 

### 4.7. Nanocarriers-Mediated Delivery of Gene- and Cell-Based Therapies

Among cell-based therapies, the use of nanocarriers in stem-cell therapy is most prominent. Polymeric nanoparticles were exploited for their potential to facilitate the transfer of genes in human embryonic stem cells. The positively charged nanocarriers of approximately 200 nm produced a fourfold increase in the transfection of cells with minimal toxicity and adverse effects [140]. Nanoparticles were prepared to carry regenerative factors from mesenchymal stem cells and were further coated with the membranes from red blood cells to enhance their blood stability. They were administered intravenously in mice with carbon tetrachloride-induced liver failure. The prepared nanoparticles not only mitigated the liver failure but also promoted the growth and proliferation of hepatic tissues [141]. Glycosaminoglycan-based hybrid hydrogel encapsulated with polyelectrolyte complex nanoparticles were studied for endogenous stem cell regulation in central nervous system regeneration [142]. Neurogenesis and angiogenesis in an ischemic stroke model were improved by the delivery of stromal-derived factor-1α and basic fibroblast factor. In addition, enhanced tissue regeneration was observed. 

Various nanoparticles have been explored in cancer stem cell therapies. PLGA nanoparticles loaded with salinomycin revealed sufficient targeting in osteosarcoma, thereby reducing the expression of CD133 [143]. Similarly, codelivery of salinomycin and paclitaxel was shown to target CD44+ cells when delivered via PLGA nanocarriers [144]. Nanoparticles targeting CD133 through conjugation with an anti-CD133 mAb were investigated against breast cancer and demonstrated significantly enhanced therapeutic efficiency compared with the control condition [145]. PEG nanocarriers loaded with bortezomib were targeted to reduce the expression of cancer stem cells and treat breast cancer. The nanocarriers sufficiently accumulated in the stem cells and enhanced the therapeutic efficiency [146]. Docetaxel PLA nanoparticles were studied for targeted delivery to lung cancer stem cells and a profound antimetastatic response was demonstrated both in vitro and in vivo [147]. Cationic albumin nanoparticles functionalized with hyaluronic acid were investigated to target cancer stem cells overexpressing CD44 [148]. The uniform-sized spherical nanoparticles demonstrated a high affinity and specific binding to CD44-enriched B16F10 cells, as well as tumor internalization in a mouse lung-tumor model, which significantly limited tumor growth and metastasis. 

Stem-cell-based therapy with nanocarriers in cardiovascular diseases is another important aspect of therapeutics. Chitosan-alginate nanoparticles were used to deliver placental growth factors, which improved cardiac functioning at the site of an acute myocardial infarction [149]. The delivery of hepatocyte growth factor genes using mesoporous organosilica nanoparticles also demonstrated enhanced paracrine activity in hepatocyte growth factor-transfected myocardial stem cells, resulting in reduced apoptosis and increased angiogenesis in a rat model of myocardial infarction [150]. Inorganic nanocarriers, particularly magnetic nanoparticles with liposomes, were found to successfully transfer human myocardial stem cells, which increased the expression of vascular endothelial growth factor and reduced the incidence of apoptosis in unilateral hind limb ischemic animal models [151].

## 5. Hurdles in the Clinical Translation and Commercialization of Nanocarriers

Nanocarrier-based delivery of biopharmaceuticals has been established as an effective alternative to traditional methods. However, lots of hurdles in the clinical translation and commercialization of these nanocarriers still remain. The development of nanocarriers is a more tedious and time-consuming process involving far more complex strategies than conventional formulations. We here present the major challenges to the successful use of nanocarriers for biopharmaceutical delivery. 

### 5.1. Biological Hurdles

Controlling the biological fate of nanocarriers inside the human body is one of the major challenges. The clinically investigated nanocarriers utilized PEGylation to enable long-term circulation in the blood without being taken up by the reticuloendothelial system [60] and ligand conjugation for targeting with antibodies such as HER2 and EGFR [164,165]. In addition, the interaction between nanocarriers and biological barriers is an important factor. Nanocarriers loaded with biopharmaceuticals have been focused on cellular internalization and the molecular interactions of the desired moiety including the enhanced permeability and retention (EPR) effect at tumor site [166]. The ability of nanocarriers to penetrate biological barriers enhances the delivery of biopharmaceuticals inside tissues [167,168]. Apart from these characteristics, differences in pathological conditions and in vivo behavior between humans and animals also reduce the clinical use of nanocarriers [169]. 

Moreover, the correlation in targeting between humans and animals can vary depending on the methods used in preclinical animal studies and human clinical studies. For example, organ extraction and tissue harvesting to confirm the in vivo behavior of biopharmaceuticals is impractical in human clinical studies [170]. Clinically, biopharmaceuticals are mainly used for hormone replacement therapy, cancer therapy, and the treatment or prevention of infectious and inflammatory diseases. Interpatient variability, target expression, and dose-dependent anatomical and pathological conditions can cause variations in biodistribution. This is another reason why nanocarriers are not commonly used in clinics despite the existence of sufficient data from animal studies [171]. 

### 5.2. Technological Hurdles

Technological challenges hindering the clinical use of nanocarriers for biopharmaceutical delivery predominantly involve the large-scale manufacturing of the formulations, the optimization of leads through high-throughput screening, and the prediction of clinical outcomes in large populations. Existing investigations of nanocarriers for biopharmaceutical delivery are mainly based on laboratory-scale preparation for assessment in animal models. Upon scale-up, the reproducibility and stability of the formulations become questionable [172]. Careful observation is required when scaling up process for large-scale manufacturing since biopharmaceuticals have sensitive moieties. Due to the lack of quality testing procedures, scalability complications, uncertain formulation stability, and funding issues, nanocarrier-based biopharmaceutical delivery continues to be investigated in animal models using laboratory procedures and has not reached the clinics [173,174]. Computational and theoretical modeling can use experimental data to predict the clinical outcomes of new formulations. Several devices and technologies that mimic biological systems can provide a better prediction of the clinical outcomes for specific nanocarriers [175]. Substantial advances can be made in the clinical use of nanocarriers by carrying out the necessary investigations for these models. 

### 5.3. Nanotoxicological Hurdles

Extensive safety and biodistribution profiles need to be compiled prior to the clinical use of nanocarriers to deliver biopharmaceuticals to humans. Specific safety assessments are needed for the chemicals used in manufacturing nanocarriers, the compatibility of biopharmaceuticals with nanocarrier components, and the process of nanocarrier development before nanocarrier-based biopharmaceuticals can move into clinical use [176,177]. The safety determinations of nanocarrier components, particularly lipids and polymers, have been conducted on multiple occasions. However, the safety profiles of synthetic components, ligands and coatings, must be considered in terms of biodistribution and toxicity upon in vivo administration [178]. The in vivo absorption, distribution, metabolism, and excretion (ADME) characteristics of nanocarriers need to be fully understood. The drug-loaded nanoparticles often possess distinct and complicated in vivo ADME profile compared with free drug. The altered disposition of nanocarriers presents new toxicity concerns, which should be evaluated to understand the relationship between exposure and efficacy. Furthermore, the unintended biological interactions of nanocarriers, chronic exposure to nonbiodegradable materials, and increased penetration into biological barriers contribute to their additional safety concerns. These variables necessitate additional ADME studies on nanocarriers to facilitate their development [179]. Physiologically based pharmacokinetic (PBPK) models can help in predicting the pharmacokinetic parameters and the risk assessment of nanocarriers [180,181]. Although in vitro, in vivo, and ex vivo studies have investigated the safety of nanocarriers in various cell lines and animal models, the biological responses in humans can vary, limiting the relevance of safety assessments in animal studies [182]. Thus, the careful early consideration for the effect of varying administration routes, the influence of biological components on drug release, and the optimal formulation methods could increase the chances of success in clinical translation. 

## 6. Conclusions

The groundbreaking success of biopharmaceuticals in recent years has revolutionized the treatment of many ailments. However, formulation and administration challenges still remain. Colloidal nanocarriers could be a promising tool to bypass these challenges. Nanotechnology not only offers new methods for biopharmaceutical synthesis but also suggests techniques for noninvasive, safe, and targeted delivery. Moreover, the accessibility of biopharmaceuticals to target sites for the treatment of specific pathological conditions could also be made convenient through the use of nanotechnology. Despite the excellent characteristics of nanocarriers, the clinical translation and commercialization for biopharmaceutical delivery remain uncertain due to biological and technological complications. Considerable efforts are required to scale up nanocarrier formulations and conduct the quality control to manage their physicochemical properties. The nanocarrier-based biopharmaceuticals involved in a particular therapy need to be assessed for efficacy and short- and long-term toxicity. Altogether, nanocarrier-based delivery of biopharmaceuticals has great potential for the effective treatment of multiple pathological conditions including cancers, autoimmune disorders, and other diseases.

## Figures and Tables

**Table 1:** Biopharmaceuticals and their clinical indications approved by the FDA in 2018–2020 [31].

Brand Name	Generic Name	Target	Class	FDA Approved Indications	Company/Developer
**Biopharmaceuticals approved in 2020**					
Tacartus	Brexucabtagene autoleucel	TNF	mAb	Mantle cell lymphoma	Kite Pharma
Hulio	Adalimumab	TNF	mAb	Rheumatoid arthritis, juvenile idiopathic arthritis, psoriatic arthritis, ankylosing spondylitis, Crohn’s disease, ulcerative colitis, and plaque psoriasis	Mylan and Fujifilm Kyowa Kirin Biopharmaceuticals
Tepezza	Teprotumumab	IGF-1R	mAb	Thyroid eye disease	Horizon Therapeutics
Phesgo	Pertuzumab, transtuzumab, and hyaluronidase	HER + hyaluronidase	mAb	Early HER-2-positive breast cancer	Genentech/Roche
Lyumjev	Insulin lispro	Beta-cells	rDNA	Type I and type II diabetes	Eli Lilly & Co.
Semglee	Insulin glargine	Beta-cells	rDNA	Type I and type II diabetes	Biocon
Uplizna	Inebilizumab	Aquaporin-4	mAb	Neuromyelitis optica spectrum disorder	Viela Bio
Nyvepria	Pegfilgrastim	Filgrastim	rDNA	Neutropenia	Neulasta
Trodelvy	Sacituzumab	Trop-2	mAb	Metastatic triple negative breast cancer	Immunomedics
Sarclisa	Isatuximab	CD38	mAb	Multiple myeloma	Sanofi-Aventis
Influenza vaccine	H1n1 influenza vaccine	Virus	Vaccine	Prevention of seasonal influenza	Seqirus
Vyepti	Eptinezumab	CGRP	mAb	Migraine	Lundbeck
Tepezza	Teprotumumab	IGF-IR	mAb	Thyroid eye disease	Horizon Therapeutics Ireland
**Biopharmaceuticals approved in 2019**					
Cutaquig	Human immunoglobulin	Immune cells	Ab	Primary humoral immunodeficiency	Octapharma Pharmazeutika
Ubrelvy	Ubrogepant	Calcitonin	rDNA	Migraine	Allergen USA
Enhertu	Trastuzumab	HER-2	mAb	Breast cancer	Astra Zeneca and Daiichi Sankyo Co. Ltd.
Ervebo	Ebola Zaire vaccine	Glycoprotein	Vaccine	Ebola disease	Merck & Co
Padcev	EnfortumAb-vedotin	Nectin-4	mAb	Urothelial cancer	Seattle Genetics
Vyondys 53	Golodersin	Dystrophin antisense	Oligonucleotide	Duchenne muscular dystrophy	Sarepta Therapeutics
Avsola	Infliximab	TNF	mAb	Autoimmune disorders	Amgen
Givlaari	Givosiran	ALN-AS1 mRNA	RNAi	Acute hepatic porphyria	Alnylam Pharmaceuticals
Adakveo	Crizanlizumab	P-selectin	mAb	Vaso-occlusive crisis	Novartis
Abrilada	Adalimumab	TNF	mAb	Rheumatoid arthritis, juvenile idiopathic arthritis, psoriatic arthritis, ankylosing spondylitis, Crohn’s disease, ulcerative colitis, and plaque psoriasis	Pfizer
Reblozyl	Luspatercept	Activin receptor-igg1	Fusion protein	Anemia with beta thalassemia	Celgene
Ziextenzo	Pegfilgrastim	G-CSF	rDNA	Neutropenia	Sandoz/Novartis
Beovu	Brolucizumab	VEGF	mAb	Neovascular (wet) age-related macular degeneration	Novartis
Bonsity-teriperatide	Parathyroid hormone	PTH	Protein	Osteoporosis	Pfenex Inc.
Jynneos	Smallpox and monkeypox vaccine	Viral proteins	Protein	Smallpox and monkeypox vaccine	Bavarian Nordic
Rybelsus	Semaglutide	Glucagon like peptide 1	Protein	Type 2 diabetes	Novo Nordisk
Hadlima	Adalimumab	TNF	mAb	Rheumatoid arthritis, juvenile idiopathic arthritis, psoriatic arthritis, ankylosing spondylitis, Crohn’s disease, ulcerative colitis, and plaque psoriasis	Samsung Bioepis
Ruxience	Rituximab	CD20	mAb	Cancer	Pfizer
Myxredlin	Insulin, human	Beta cells	Glycoprotein	Diabetes	Baxter
Baqsimi nasal powder	Glucagon	-	rDNA	Hypoglycemia	Eli Lilly & Co.
Xembify	Immunoglobulin subcutaneous	Immune cells	Ab	Primary immunodeficiency	Grifols
Zirabev	Bevacizumab	VEGF	mAb	Colorectal cancer, nonsquamous nonsmall cell lung cancer, glioblastoma, metastatic renal cell carcinoma, and cervical cancer	Pfizer
Kanjinti	Trastuzumab	HER-2	mAb	HER2-positive breast cancer and gastric cancer	Amgen
Polivy	Polatuzumab	CD79b	mAb	Diffuse large B-cell lymphoma	Genentech/Roche
Zolgensma	Onasemnogene-abeparvovec	Survival motor neuron 1	Gene therapy	Spinal muscular atrophy	AveXis
Dengvaxia	Dengue tetravalent vaccine	Viral protein	Vaccine	Dengue disease	Sanofi Pasteur
Enticovo	Etanercept	Tnfr-Fc	Fusion protein	Rheumatoid arthritis, ankylosing spondylitis, plague psoriasis, psoriatic arthritis, and polyarticular juvenile idiopathic arthritis	Samsung Bioepis
Skyrizi	Risankizumab	IL-23	mAb	Plaque psoriasis	AbbVie
Evenity	Romosozumab	Sclerostin	mAb	Osteoporotic fracture	Amgen
Asceniv	Immunoglobulin	IVIG	Ab	Primary humoral immunodeficiency disease	ADMA Biopharmaceuticals
Trazimera	Trastuzumab	HER receptor	mAb	Breast cancer	Pfizer
Herceptin hylecta	Trastuzumab and hyaluronidase	MAb plus hyaluronidase	mAb	Breast cancer	Genentech/Roche
Esperoct	Turoctocog alfa pegol	Factor VIII	Glycoprotein	Hemophilia	Novo Nordisk
Cablivi	Caplacizumab	Von Willebrand’s factor	mAb	Thrombotic thrombocytopenic purpura	Ablynx
Jeuveau	Prabotulinumtoxin toxin type A	Botulinum toxin A	Protein	Glabellar lines	Evolus Inc.
Ontruzant	Trastuzumab	HER receptor	mAb	Breast cancer	Samsung Biopharmaceuticals
**Biopharmaceuticals approved in 2018**					
Aimovig	Erenumab	CGRP	mAb	Migraine prevention	Amgen
Retacrit	Epoeitin alfa	EPO	Glycoprotein	Anemia related indication	Hospira/Pfizer
Crysvita	Trastzumab	FGF	mAb	X-linked phosphatemia	Ultragenyx Pharmaceutical Inc,
Ilumya	Tildrakizumab	IL-23	mAb	Plaque psoriasis	Sun pharmaceutical Industries LTD.
Trogarz	Ibalizumab	Cd4	mAb	HIV infection	TaiMed Biopharmaceuticals
Vaxelis	DTaP-Hb, rDNA	Protein	Hexavalent vaccine	Diphtheria, tetanus, acellular pertussis, polio virus, Hemophilus b conjugate, andhepatitis B	Sanofi Pasteur
Ultomiris	Ravulizumab	C5	mAb	Paroxysmal nocturnal hemoglobinuria	Alexion Pharmaceutical
Elzonris	Tagraxofusp-erzs	CD 123	mAb	Blastic plasmacytoid dendritic cell neoplasm	Stemline Therapeutics
Asparlas	Calaspargase	Asparaginase	Enzyme	Acute lymphoblastic leukemia	Servier Pharmaceuticals LLC
Herzuma	Transtuzumab	HER receptor	mAb	Breast cancer	Celltrion and Teva
Cutaquig	Immunoglobulin subcutaneous	Immunoglobulin	Ab	Primary humoral immunodeficiency	Octapharma
Truxima	Rituximab	CD20	mAb	Non-Hodgkin lymphoma	Celltrion
Gamifant	Emapalumab	Interferon gamma	mAb	Hemophagocytic lymphohistiocytosis	Novimmune SA
Udenyca	Pegfligrastim	G-CSF	rDNA	Neutropenia from cancer treatment	KBI Biopharma
Hyrimoz	Adalimumab	TNF	mAb	Rheumatoid arthritis, juvenile idiopathic arthritis, psoriatic arthritis, ankylosing spondylitis, Crohn’s disease, ulcerative colitis, and plaque psoriasis	Sandoz/Novartis
Revcovi	Elapegademase	Adenosine deaminase	rDNA	Adenosine deaminase-severe combined immunodeficiency	Leadiant Biosciences
Libtayo	Cemiplimab	PD-1	mAb	Cutaneous squamous cell carcinoma	Regeneron Pharmaceuticals
Emgality	Galcanezumab	CGRP	mAb	Migraine	Eli Lilly & Co.
Ajovy	Fremanezumab	CGRP	mAb	Migraine	Teva
Lumoxiti	Moxetumomab	CD22	mAb	Hairy cell leukemia	Astra Zeneca
Jivi	Anti-hemophilic factor	Factor VIII	RNAi	Hemophilia A	Bayer Corp
Takhzyro	Lanadelumab	Kallikrein	mAb	Type I and II hereditary angioedema	Dyax Corp. Shire plc
Oxervate	Cenegermin	Transthyretin	RNAi	Neurotrophic keratitis	Alnylam Pharmaceuticals
Onpattro	Patisiran	Transthyretin mRNA	RNAi	Polyneuropathy	Alnylam Pharmaceuticals
Poteligeo	Mogamulizumab	CCR-4	mAb	Resistant mycosis fungoides or Sezary syndrome	Kyowa Kirin
Panzyga	Immunoglobulin intravenous	Immune cells	Ab	Immune thrombocytopenic purpura	Octapharma
Nivestym	Filgrastim	G-CSF	rDNA	Neutropenia	Pfizer
Human albumin solution	Albumin	-	Albumin	Hypovolemia, ascites, hypoalbuminemia, acute nephritis, and cardiopulmonary bypass	Bio Products Library
Fulphila	Pegfilgrastim	G-CSF	rDNA	Neutropenia	Mylan GmbH
Palynziq	Pegvaliase	Phenylalanine ammonia lyase	rDNA	Phenylketonuria	BioMarin

**Abbreviations:** TNF: tumor necrosis factor; mAb: monoclonal antibody; IGF-1R: insulin-like growth factor 1 receptor; HER: human epidermal growth factor; rDNA: recombinant deoxyribonucleic acid; Trop II: trophoblast self-surface antigen 2; CD: cluster of differentiation; G-CSF: granulocyte colony-stimulating factor: VEGF: vascular endothelial growth factor; PTH: parathyroid hormone; Tnfr-Fc: tumor necrosis factor receptor; IL: interleukin; IVIG: intravenous immunoglobulin; CGRP: calcitonin gene-related peptide; EPO: erythropoietin; FGF: fibroblast growth factor; CCR-4: C-C chemokine receptor type 4; PD-L1: programmed death-ligand 1; DTaP-IP: diphtheria and tetanus toxoids and acellular pertussis adsorbed and inactivated poliovirus.

**Table 2:** Applications of nanocarriers in biopharmaceutical delivery.

Biopharmaceuticals	Therapeutic Class	Target Disease	Nanocarrier	Route	Purpose of the Study	Characteristics of Nanocarriers	Key Findings	Reference
Insulin	Hormone	Diabetes mellitus	FA-PEG-PLGA NPs	Oral	Improving oral delivery of insulin	PS: ~260 nm, PDI: 0.14 ± 0.04, EE: 87.0 ± 1.92%	Twofold increase in insulin bioavailability following NP administration, along with maintenance of blood glucose levels for 24 h.	[69]
hGH	Hormone	Hormone deficiency	Thermosensitive hydrogel	Subcutaneous	To enhance the bioavailability and sustained release of hGH	PS: 500 nm, ZP: +8 mV	Sustained release of hGH for 7 days, with a 13-fold extended half-life in hypophysectomized rats.	[71]
rhGH	Hormone	Hormone deficiency	Dextran NPs	In vitro assay on rat Nb_2_-11 lymphoma cells	Efficient and stable rhGH delivery	PS: ~25 nm	99% bioactivity of rhGH was preserved and analyzed by Nb_2_-11 cell proliferation assay.	[152]
Melatonin	Hormone	Depression	PLA-NPs	Subcutaneous	Enhancing the antidepressant activity and HPA hormone modulation of melatonin	PS: 96.1 ± 13.5 nm, PDI: 0.203 ± 0.01 EE: 33.82 ± 0.53%	Pharmacodynamic models, sucrose preference test, FST, and TST demonstrated efficient antidepressant activity, and HPA axis hormone secretion in pinealectomized rats also improved.	[75]
Estradiol	Hormone	Osteoporosis	PLGA-NPs	Transdermal	Increasing skin permeability of estradiol using a nanocarrier and iontophoresis	PS: 165 ± 13.1 nm, EE: 63.4 ± 3.09%	Bone mineral density was significantly increased after iontophoresis; permeation of estradiol also increased, with an effective concentration in blood.	[153]
IFNα-2b	Cytokines	Cancers and viral infections	Chitosan NPs	Oral	To improve oral delivery of IFN	PS: 36 ± 8 nm, ZP: +30 mV EE: ~100%	Antiviral activity of NPs in vitro and IFN gene expression were comparable to commercial IFNα; remarkable plasma levels of IFNα were observed following oral administration in mice.	[84]
IL-2	Cytokines	Immune therapy	Nanocapsules	Intravenous	To enhance T cell-based immune therapy by IL-2	PS: 215 nm, ZP: −7 mV	In vitro T cell targeting and in vivo IL-2 receptor-mediated internalization were enhanced.	[154]
TGF-b and IL-2	Cytokines	Cancer and autoimmune diseases	PLGA NPs	Intraperitoneal	Induction and maintenance of Treg cells by CD4 targeted nanoparticles	PS: 168 nm	In vitro induction and in vivo expansion of CD4+ Treg cells was observed.	[155]
IL-4	Cytokines	Immune therapy	MSNs	Intraperitoneal	Macrophage polarization by cytokine delivery	PS: <200 nm	Targeted delivery of cytokines to phagocytic myeloid cells triggering macrophage polarization and the induction of an immune response.	[86]
IL-15	Cytokines	ACT in metastatic tumors	Nanogels	Intravenous	To enhance T cell therapy through TCR signaling	PS: 80–130 nm, EE: >90%	A 16-fold increase in T cell expansion was observed in tumor cells; increased tumor cell clearance in mice.	[156]
siRNA	Nucleotide	Gene therapy in cancers	HAS-NPs	In vitro assay in MCF-7 cells	To prevent degradation and low transfection of siRNA	PS: ~90 nm, ZP: +26 mV, PDI: <0.25	High transfection (61.66 ± 6.8%) and cytotoxicity were observed.	[157]
siRNA	Nucleotide	Intestinal inflammation	PLGA-PEI-NPs	Intrarectal	To prevent intestinal inflammation by colonic gene silencing	PS: 151.52 nm, PDI: 0.38, ZP: 22.08 mV	Excellent gene silencing with no toxicity in cell culture; in vivo application resulted in significant decrease in the target genes in colonic biopsies and mesenteric lymph nodes.	[92]
CD98 siRNA	Nucleotide	Nonalcoholic fatty liver disease	PLA-NPs	Parenteral	To reduce hepatic steatosis in mice	PS: 280 nm, ZP: −12.84 mV	Significant downregulation of CD98 and pro-inflammatory cytokines was observed, along with a reduction in blood markers, lipid accumulation, and fibrosis in vivo.	[93]
CD73-specific siRNA	Nucleotide	Breast cancer	Chitosan lactate NPs	Intravenous	To evaluate anti-angiogenic effects of CD73 suppression	PS: 70–126 nm, PDI: ~0.3, ZP: ~19 mV, EE: 50–90%	Downregulation of angiogenesis-related molecules and pro-inflammatory cytokines, along with tumor regression due to CD73 gene silencing.	[94]
HBcAg antigen	Vaccine	Hepatitis B	PLGA-NPs	Subcutaneous	To enhance the immune response against hepatitis B virus	PS: 279 nm, PDI: 0.17, EE: ~50%	Cellular immune response with high TNF-γ.	[109]
Recombinant Ebola virus antigen	Vaccine	Ebola virus disease	Lipid NPs	Subcutaneous	To induce potent antibody and polyfunctional T cell responses	PS: 117.5 ± 17.6 nm, PDI: 0.18 ± 0.01, ZP: −21.7 ± 1.3 mV, EE: ~60%	Germinal center B cells and polyfunctional T cells were produced, along with elicited antibody response.	[110]
*H. pylori* recombinant antigen	Vaccine	Peptic ulcer	PLGA-NPs	Oral	Increasing immune protection in *Helicobacter pylori* infections	PS: ~200 nm, PDI: 0.228 ± 0.030, EE: 79.07%	43% of the immunized mice showed a protective effect from infection, along with high levels of urease-specific antibodies and memory T cell responses.	[111]
Ovalbumin	Vaccine	Immune therapy	Calcium phosphate NPs	Oral	Enhancing oral vaccine efficacy	PS: 22 nm, ZP: −9.6 mV	Sufficient GI stability, along with effective Caco-2 permeability and enhanced IgA and IgG responses.	[158]
HPV antigen	Vaccine	Cervical cancer	VLPs	Oral for systemic and vaginal for local action	Combining the effects of VLP- and DNA-based vaccines	–	Induction of antibody and T cell response.	[159]
mRNA-based vaccines	Vaccine	Immune therapy	Lipid NPs	Intravenous	Efficient transport of mRNA-based cancer vaccines	PS: 110 nm, ZP: 25 mV, EE: 80%	Strong and specific T cell response and reduced tumor growth in lymphoma model.	[115]
mRNA-based vaccines	Vaccine	HIV	PLA-NPs	In vitro and ex vivo assay	Targeting dendritic cells for effective immune responses	PS: ~275 nm, PDI: 0.13, ZP: 30 mV	Effective phagocytic uptake with strong induction of dendritic cells.	[116]
Cancer antigens	Vaccine	Tumor	MSNs	Subcutaneous	To deliver large amounts of protein antigen and Toll-like receptor 9 agonist for enhanced cancer vaccine efficacy	PS: 100–200 nm, ZP: −10.5 mV	Efficient delivery of TLR9 agonist to draining lymph nodes, induction of antigen-specific cytotoxic T lymphocytes, and suppression of tumor growth.	[160]
Tn antigen	Vaccine	Tumor	Dextran-based NPs	Ex vivo assay	To conjugate synthetic Tn-antigen mimetic to dextran-based single-chain nanoparticles	PS: ~70 nm, PDI: 0.4, ZP: −18.8 mV	Specific innate tumor modulation, as demonstrated by analysis of IL production.	[161]
Infliximab	Antibody	Autoimmune uveoretinitis	Liposomes	Intravitreal	To evaluate the effectiveness of intravitreal injection of liposomes encapsulating infliximab.	PS: 351.3 ± 58 nm, EE: 90.65 ± 2.68%, PDI: 0.386 ZP: −20.8 ± 9.8 mV	Decreased inflammation in eyes with lower toxicity and side effects in autoimmune uveoretinitis rats.	[120]
1E4-1C2 mAb	Antibody	Hepatocellular carcinoma	Chitosan NPs	In vitro mouse monocyte models	Improving the delivery of mAbs against hepatocellular carcinoma	PS: 11.2 ± 0.09 nm, ZP: 16.5 ± 0.5 mV	Sufficient cellular uptake by mononuclear cells and reduced cytotoxicity in monolayer cells.	[122]
Anti-HER2 mAb	Antibody	Cancers	PEGylated HSA NPs	In vitro assays	Improving the delivery of anti-HER2 mAbs to cancers	PS: 203 ± 15 nm, PDI: 0.07 ± 0.02, ZP: −14.2 ± 2.1 mV	High interaction with HER2 receptors on the surface of BT474 cells, with no noted toxicity.	[124]
Cetuximab	Antibody conjugation	Nonsmall cell lung cancer	PLGA-NPs	Intravenous	Bioconjugation of cetuximab with paclitaxel to enhance its efficacy	PS: 80 nm, ZP: −50 mV, EE: 85–100%	High binding affinity toward overexpressed EGFR cells in tumors; in mice, high inhibition of tumor growth and increased survival rate.	[125]
Rituximab	Antibody conjugation	Leukemia	PLGA-NPs	Subcutaneous	Targeted delivery of Nutlin-3 toward CD20 malignant cells using antibody conjugated nanocarriers	–	Increase in the activation of the p53 pathway and enhanced tumor suppression.	[162]
Transferrin and 2C5 mAb	Antibody conjugation	Ovarian cancer	Micelles	Subcutaneous	To increase cytotoxicity and targeting efficiency of poorly water-soluble anticancer drug	PS: ~16 nm	In vitro cytotoxicity against ovarian cancer cells was optimal, along with targeted and profound in vivo antitumor activity due to antibody conjugation.	[126]
EGFR-targeted mAb	Antibody conjugation	Epidermoid carcinoma tumor	Au-NPs	Intravenous	To enhance tumor targeting and biodistribution	PS: ~5 nm Antibody loading: 1.7 nmol/mg	Enhanced biodistribution profile in both in vitro and in vivo carcinoma models.	[127]
Trastuzumab- and Fab′ fragment	Antibody conjugation	Breast cancer	PEG-PLGA NPs	Intravenous	Targeted delivery of curcumin nanoparticles to HER2 in breast cancer cells	PS: 128.5 ± 1.3 nm and 142.5 ± 4.6 PDI: 0.125 ± 0.012 and 0.137 ± 0.023 ZP: 79.5 ± 1.56 and 77.1 ± 5.64 mV	Enhanced cytotoxicity against HER2 cells in vitro and enhanced biodistribution in vivo.	[163]
Cysteine proteinase type-I	Enzyme	Leishmania major infection	SLNs	Intraperitoneal	To develop safe, immunogenic vaccine against Leishmania with potent immune response	PS: 380 nm, PDI: 0.4, ZP: −12·4 ± 0·3 mV EE: 48 ± 3%	Following vaccination, the occurrence of parasite decreased, and the cytokine response increased, indicating the necessary immune response.	[135]
Tissue plasminogen activator	Enzyme	Subconjunctival hemorrhages	Liposomes	Intravenous	Enhancing the thrombolytic activity of tissue plasminogen activator	PS: 600 nm, EE: 50%	Thrombolytic activity was sufficient and comparable to other clinical regimens.	[137]
Streptokinase	Enzyme	Deep vein thrombosis	Chitosan NPs	In vitro assay	Developing streptokinase-loaded nanocarriers for efficient thrombolytic activity	PS: 526 ± 121 nm, PDI: 0.3 ± 0.2, EE: 43 ± 10%	Thrombolytic activity was sufficient in vitro, along with lack of cytotoxic activity.	[138]
Streptokinase	Enzyme	Thrombosis	Liposomes	Intraarterial	To estimate the effect of RGD peptide conjugation on the biodistribution behavior of liposomes	PS: 115 ± 12 nm, PDI: 0.158 ± 0.043 EE: 18.0 ± 1.3%	Thrombolytic activity was sufficient, with increased accumulation in the thrombus.	[139]
Mesenchymal stem cells	Gene- and cell-based therapy	Acute liver failure	PLGA-NPs	Intravenous	To enhance therapeutic efficacy and increase tolerability	PS: 200 nm, ZP: −10 mV	Increased internalization and growth of liver cells.	[141]
Salinomycin	Gene- and cell-based therapy	Osteosarcoma	PLGA-NPs	Subcutaneous	Increasing aqueous solubility and tumor targeting	PS: 150 nm, EE: 50%	CD133+ osteosarcoma was resolved both in vitro and in vivo.	[143]
Bortezomib	Gene- and cell-based therapy	Breast cancer	PLA-NPs	Intravenous	To enhance therapeutic effectiveness of bortezomib	PS: 112.8 ± 2.3 nm PDI: 0.13 ± 0.1, EE: 72.8%	Increased targeting and tumor suppression.	[146]
Placental growth factor	Gene- and cell-based therapy	Myocardial infarction	Chitosan alginate NPs	Intramyocardial	Sustained release and prolonged effect of placental growth factor	PS: 100–200 nm, ZP: 7.2 ± 0.5 mV, EE: 38.4% ± 3.4%	Significant increase in cardiac functioning, with decreased incidence of inflammation and negligible toxicity.	[149]
Mesenchymal stem cells	Gene- and cell-based therapy	Myocardial infarction	MSNs	Intramyocardial	To overcome toxicity and insufficient gene transfection.	PS: 514 nm	Decrease in apoptotic cardiac myocytes, reduced infarct and fibrosis, increased angiogenesis.	[150]
Mesenchymal stem cells	Gene- and cell-based therapy	Ischemia	Magnetite NPs in liposomes	Parenteral	To enhance the targeting of ischemic tissues	PS: 10 nm	Enhanced therapeutic activity in ischemia-induced angiogenesis.	[151]

**Abbreviations:** PS: particle size; ZP: zeta potential; PDI: polydispersity index; EE: entrapment efficiency; SLNs: solid lipid nanoparticles; NPs: nanoparticles; PLGA: poly(d,l-lactic-co-glycolic) acid; FA: folate; Au: gold; hGH: human growth hormone; IFN: interferon; IL: interleukin; MSNs: mesoporous silica nanoparticles; ACT: adoptive cell therapy; siRNA: small interfering ribonucleic acid; HSA: human serum albumin; VLPs: virus-like particles; Tn: tumor associated carbohydrate; HPV: human papilloma virus; mRNA: messenger ribonucleic acid.
